# Feasibility of Rapid Diagnostic Technology for SARS-CoV-2 Virus Using a Trace Amount of Saliva

**DOI:** 10.3390/diagnostics11112024

**Published:** 2021-11-01

**Authors:** Reiko Tokuyama-Toda, Masaaki Muraoka, Chika Terada-Ito, Shinji Ide, Toshikatsu Horiuchi, Tsuyoshi Amemiya, Airi Fukuoka, Yoshiki Hamada, Shunsuke Sejima, Kazuhito Satomura

**Affiliations:** 1 Department of Oral Medicine and Stomatology, School of Dental Medicine, Tsurumi University, 2-1-3, Tsurumi, Tsurumi-ku, Yokohama City 230-8501, Japan; tokuyama-r@tsurumi-u.ac.jp (R.T.-T.); terada-chika@tsurumi-u.ac.jp (C.T.-I.); ide-shinji@tsurumi-u.ac.jp (S.I.); 2 Certified Non-Profit Organization Biomedical Science Association, 2-20-8, Kamiosaki, Shinagawa-ku, Tokyo 141-0021, Japan; yosami.muraoka@gmail.com (M.M.); sejima@npo-bmsa.org (S.S.); 3 Department of Oral and Maxillofacial Surgery, Saiseikai Yokohamashi Tobu Hospital, 3-6-1, Shimosueyoshi, Tsurumi-ku, Yokohama City 230-8765, Japan; t_horiuchi@tobu.saiseikai.or.jp (T.H.); t_amemiya@tobu.saiseikai.or.jp (T.A.); a_fukuoka@tobu.saiseikai.or.jp (A.F.); 4 Department of Oral and Maxillofacial Surgery, School of Dental Medicine, Tsurumi University, 2-1-3, Tsurumi, Tsurumi-ku, Yokohama City 230-8501, Japan; hamada-y@tsurumi-u.ac.jp

**Keywords:** COVID-19, SARS-CoV-2, mobile qPCR device, mouthwash, saliva

## Abstract

Containment of SARS-CoV-2 has become an urgent global issue. To overcome the problems of conventional quantitative polymerase chain reaction (qPCR) tests, we verified the usefulness of a mobile qPCR device that utilizes mouthwash to obtain a saliva sample with the aim of developing a rapid diagnostic method for SARS-CoV-2. First, we examined whether anyone could easily operate this device. Then, we examined whether RNA in the mouthwash could be detected in a short time. In addition, we investigated whether it was possible to diagnose SARS-CoV-2 infection using mouthwash obtained from COVID-19 patients undergoing hospitalization. The results revealed that all subjects were able to complete the operation properly without error. In addition, RNase P was detected in the mouthwash without pretreatment. The average detection time was 18 min, which is significantly shorter than conventional qPCR devices. Furthermore, this device detected SARS-CoV-2 in the mouthwash of a COVID-19 patient undergoing hospitalization. The above findings verified the efficacy of this diagnostic method, which had a low risk of infection, was technically simple, and provided stable results. Therefore, this method is useful for the rapid detection of SARS-CoV-2.

## 1. Introduction

COVID-19, which is caused by the SARS-CoV-2 virus, was first discovered in 2019, and has emerged as a worldwide pandemic; therefore, its containment has become a serious and urgent global issue. For the definitive diagnosis of SARS-CoV-2 infection, detection of viral RNA by quantitative polymerase chain reaction (qPCR) is currently the most reliable and widely used method [1,2,3]. However, since the qPCR test must be performed by a skilled specialist or clinical laboratory technician, securing qPCR test equipment and skilled human resources to perform the qPCR test is an important practical issue. In addition, the long turnaround time of the current qPCR test is also a problem. These negative characteristics are associated with a decreased demand for these kinds of tests [4]. Furthermore, an adequate number of skilled human resources are needed to perform the specialized techniques, such as nasal swabs, that are required for sample collection. Therefore, a necessary step toward effective containment of COVID-19 is to expand qPCR testing for SARS-CoV-2 that entails securing skilled technicians who can perform qPCR tests and operate qPCR test equipment. Aligned with these objectives is the development of a new qPCR test method that can obtain diagnostic results in a much shorter time using samples that can be collected more easily and safely by anyone, including the tested person.

Recently, a small, portable mobile qPCR device (PCR1100, Nippon Sheet Glass Co., Ltd., Tokyo, Japan) was developed for research purposes (Supplementary Materials). PCR1100 is compact and lightweight with a main body size of H200 × W100 × D50 mm and a weight of approximately 560 g. It obtains accurate measurements within 10 min by qPCR. These results indicate that the time required for qPCR testing in clinical practice can be significantly reduced. PCR1100 successfully detected the presence of each target microorganism in food hygiene tests for *Salmonella*, *Campylobacter*, and *Escherichia coli* O-157, and in environmental bacteria tests for *Legionella*. If these detection capabilities could be achieved by a device that can be operated easily by the user, such as performing rapid detection of pathogens from saliva samples without pretreatment, then this device can be applied as a point-of-care testing (POCT) device for COVID-19 diagnosis. Furthermore, a POCT device would enable earlier diagnosis of SARS-CoV-2 infection compared to conventional qPCR methods.

Therefore, in this study, we examined whether this device could be adapted for the medical field and whether saliva samples could be used with this device for rapid diagnosis. To prove whether rapid diagnosis of SARS-CoV-2 is possible, synthetic RNA was used to confirm the diagnosis. Furthermore, we investigated whether SARS-CoV-2 viral RNA could be detected by this device using samples from COVID-19 patients who had been diagnosed as SARS-CoV-2 positive via the conventional qPCR method.

## 2. Materials and Methods

### 2.1. Sample

Positive control RNA samples of SARS-CoV-2 developed following the National Institute of Infectious Diseases (NIID) protocol were kindly provided by the NIID (for N primer probe Ver.2 and N2 primer probe Ver.2/Ver.3). Following the Centers for Disease Control and Prevention (CDC) protocol, RNA samples were synthesized by FASMAC Co., Ltd., (Atsugi, Kanagawa, Japan) based on each primer sequence and information obtained from GenBank™: accession number MN997409.1, position −10/+110 for 2019-nCoV_N1 and position +871/+980 for _N2. RNase P were similarly synthesized; accession number U77665.1, position +21/+130. Applied to each positive control, RNA was 10-fold serially diluted with 10 mM Tris-HCl (pH 8.0, molecular grade) including 10 µg/mL carrier RNA (ribonucleic acid from baker’s yeast, Merck KGaA, Darmstadt, Germany).

### 2.2. Primer and Probe

Based on previously reported protocols for SARS-CoV-2 (CDC 2020 protocol [5]), oligonucleotide primers and probes for quantitative reverse transcriptase polymerase chain reaction (qRT-PCR) detection were selected to target each N gene and were then synthesized by Nihon Gene Research Laboratories Inc. (Sendai, Japan) (Table 1). Based on the CDC protocol (CDC 2020), oligonucleotide primers and probes to detect human RNase P were also synthesized.

### 2.3. Reverse Transcription-Polymerase Chain Reaction

The PCR1100 device was used to perform one-step RT-PCR for all tests in this study. Briefly, the composition of the qRT-PCR reagent was described, 3 µL of a sample (raw sample or positive control RNA) was amplified in a 20 µL reaction solution containing 1× reaction buffer, 0.25, 0.50, or 1.0 µL of RT Enzyme Mix, 1.0 µL DNA polymerase (THUNDERBIRD™ Probe One-Step qRT-PCR Kit, TOYOBO Co., Ltd., Osaka, Japan), and a final concentration of primer/probe for each target (Table 1). For the preliminary tests, each composition was optimized. As a result, the adequate volume for the RT Enzyme Mix was 0.25 µL for the CDC protocol.

The qRT-PCR conditions that were adequate for the PCR1100 device used in this study were programmed as follows: RT incubation and enzyme activation were serially performed at 50 °C for 150 s and 95 °C for 15 s. Afterwards, it was cycled 50 times 95 °C for 3.5 s for denaturation. Annealing/amplification were performed as follows: 60 °C for 16 s for the NIID protocol; 60 °C for 7 s for the CDC protocol; 60 °C for 8 s for HCoV-229E; 60 °C for 8 s for multichannel detection of SARS-CoV-2 and RNase P following the CDC protocol. For the preliminary tests, each condition was optimized. The procedure for the PCR1100 device was conducted according to previously reports [6].

### 2.4. User-Friendliness of PCR1100

**Figure 1:**
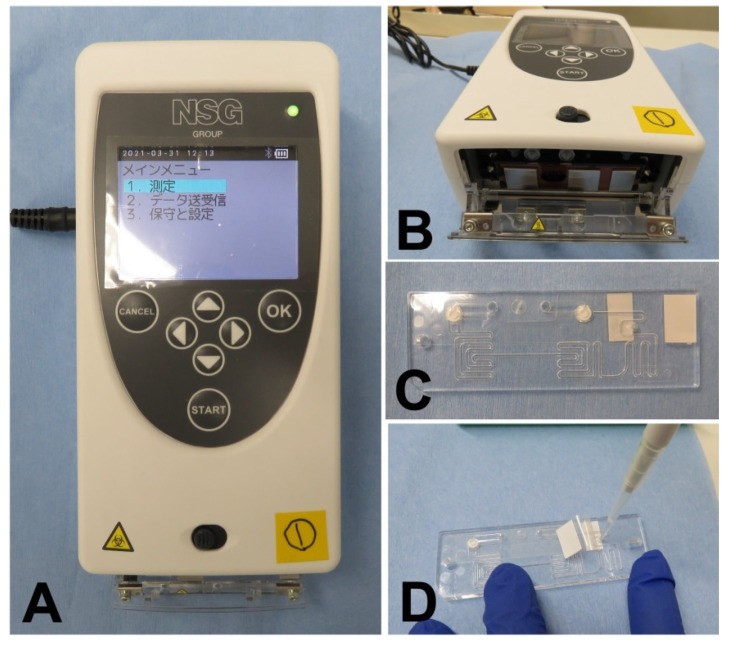
PCR1100 device body and flow path chip: (**A**) PCR1100 body (top). (**B**) Bottom (opening). Three heat blocks were provided. (**C**) Flow path chip. The sample moved in the flow path, the temperature was controlled by three heat blocks, and the PCR reaction proceeded. (**D**) The sample was injected into the hole of the flow path tip.

To achieve the aim of this study, it was necessary to obtain a diagnosis result in a short time using a technique that can be performed easily. Therefore, a commercially available one-step qRT-PCR reagent was adopted to ensure that it could be obtained easily. Verification was conducted under the approval of the Ethics Review Committee of the Faculty of Dentistry, Tsurumi University, Japan (No. 1834). First, 10 volunteers (Table 2), including nurses and dental hygienists who did not have expertise in qPCR testing, were recruited to confirm that the PCR1100 device was easily available by any healthcare professional. After a brief 10 min explanation of the procedure, it was examined whether it was possible to perform the qPCR test using the PCR1100. Since the purpose of this study was to confirm that there were no technical hurdles when performing qPCR using this device, RNase P synthetic RNA, which is widely used as an endogenous control, was added to purified water for use as a sample. The primers and probes that are specific for RNase P, which are shown in Table 1, and the THUNDERBIRD^®^ Probe One-Step qRT-PCR Kit (TOYOBO), which is a one-step qRT-PCR reagent, were prepared and mixed for the premix solution (20 µL). Using these steps, the procedure performed by the examinees was limited to mixing the premix and the sample, and verification was performed by focusing on the application of the sample to the flow path chip and determining the ease of handling of the device. The examinees were first given a brief explanation of how to handle the pipette. Then, 1 µL of the sample was mixed with the premix to prepare a reaction solution, and 16.5 µL of the reaction solution was collected and applied to the flow path chip. A pre-set reaction program was selected; the flow path chip was placed in a predefined position; and the reaction was initiated. Then, it was verified whether the device operated normally (Figure 1).

### 2.5. Possible Usage of a Trace Amount of Saliva without Pretreatment as a Sample

Verification was conducted under the approval of the Ethics Review Committee of the Faculty of Dentistry, Tsurumi University, Japan (No. 1841). The purpose of this examination was to investigate whether it was possible to prepare saliva without pretreatment of the sample for qPCR performed by this device. First, saliva was collected from healthy volunteers, and 1 µL of the undiluted solution and the 2-fold diluted solution were used as samples to examine whether the mixed RNase P synthetic RNA could be detected. In addition, it was also examined whether saliva could be used universally as a sample by mixing the saliva of five volunteers to produce a representative saliva.

In this study, we investigated whether it was possible to detect RNase P originally contained in saliva without mixing synthetic RNA into the sample saliva. Saliva collected from volunteers was diluted 2-fold and 4-fold with phosphate-buffered saline (PBS), of which 1 µL was used as a sample to examine whether RNase P could be detected by the PCR1100.

### 2.6. Possible Usage of Mouthwash as a Sample Instead of Saliva

In this study, we investigated whether it was possible to use mouthwash as a sample, as it would be easier to collect a sample of mouthwash than a sample of saliva itself. To collect the sample, after gargling the mouth thoroughly with 1 or 2 mL of diluted PBS or saline for 10 or 15 s, mouthwash was spat out into a clean tube. We investigated whether RNase P could be detected using 3 µL of this mouthwash. In addition, 3 µL of the mouthwash produced from washing the mouth with 2 mL of physiological saline for 15 s was examined as a sample. Furthermore, since it may not be possible to use the collected sample immediately for qPCR testing in clinical practice, we decided to confirm whether endogenous RNA could be detected even when the mouthwash had been stored frozen for 2 weeks.

### 2.7. Influence of Sample Collection Conditions on qRT-PCR

The timing of collecting mouthwash was also examined. It was investigated whether it was possible to collect mouthwash under three conditions: normal conditions of ~3 h after eating and brushing teeth, 30 min after lightly washing the mouth after eating, and 15 min after brushing teeth, and we used these as samples.

### 2.8. Detection of SARS-CoV-2 Synthetic RNA in Mouthwash Sample

Next, we investigated whether SARS-CoV-2 RNA could be detected using the PCR1100. Synthesized SARS-CoV-2 RNA was mixed with the mouthwash collected under the three test conditions, and it was examined whether it could be detected by the PCR1100. A premix solution was prepared by mixing the two qPCR primers and probes used for SARS-CoV-2 detection according to the CDC protocol (2019-nCoV_N1, 2019-nCoV_N2) (Table 1) and the qRT-PCR reagent (20 µL). Then, we mixed 3 µL of mouthwash containing SARS-CoV-2 synthetic RNA with the premix and performed qPCR to examine whether SARS-CoV-2 RNA could be detected by the PCR1100. In addition, to prevent degradation of RNA by saliva, a sample in which synthetic RNA was first mixed with premix was also verified.

### 2.9. Detection of SARS-CoV-2 Viral RNA in Mouthwash Obtained from COVID-19 Patients

Finally, we investigated whether this diagnostic method could be used to detect COVID-19 in patients who had been diagnosed as SARS-CoV-2 positive by the conventional qPCR test. Using the mouthwash of a COVID-19 patient as a sample, we investigated whether SARS-CoV-2 RNA could be detected with this device. This verification was conducted under the approval of the Tsurumi University School of Dentistry Ethics Review Committee (No. 121002) and the Saiseikai Yokohamashi Tobu Hospital Ethics Review Committee (No. 20210012). Among patients admitted to Saiseikai Yokohamashi Tobu Hospital under the diagnosis of COVID-19, 20 adult men and women consented to participate in this study. After admission with a COVID-19 diagnosis based on conventional qPCR testing, an initial PCR1100 test was performed, followed by several additional PCR1100 tests to evaluate progression. We investigated whether the PCR1100 test could detect SARS-CoV-2 viral RNA contained in mouthwash. In addition, we investigated whether SARS-CoV-2 viral RNA detected by several PCR1100 tests during hospitalization was correlated with the individual symptom course, recovery status, and during the period after onset of symptoms.

## 3. Results

### 3.1. Analytical Limits of Detection (LoD)

**Figure 2:**
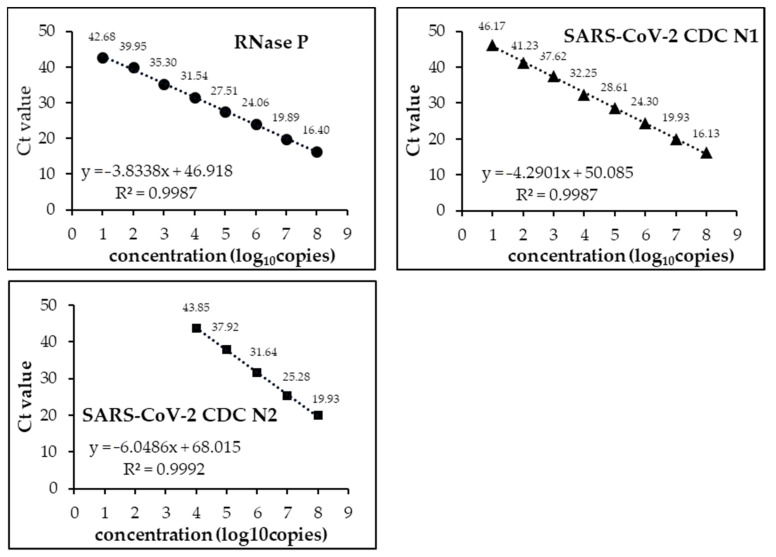
The correlation of each concentration of synthetic RNA and Ct value when the PCR1100 device was utilized to detect with multichannel. The Ct value against each concentration is demonstrated by the number above each symbol. x: log_10_ copies; y: Ct value.

We developed a multichannel system that could fully detect the targets of the CDC protocol. As shown in Figure 2, in each of RNase P, SARS-CoV-2 CDC N1, and SARS-CoV-2 CDC N2, there was a high correlation between the concentration of synthetic RNA and the Ct value. The limits of detection (LoD) were 10 copies for RNase P and SARS-CoV-2 CDC N1 but 10,000 copies for SARS-CoV-2 CDC N2. Although it may be possible to set more appropriate conditions in a multi-channel system, even in the present system, multi-channel detection using PCR 1100 clearly showed a sufficiently high sensitivity and specificity.

### 3.2. PCR1100 User-Friendliness

**Figure 3:**
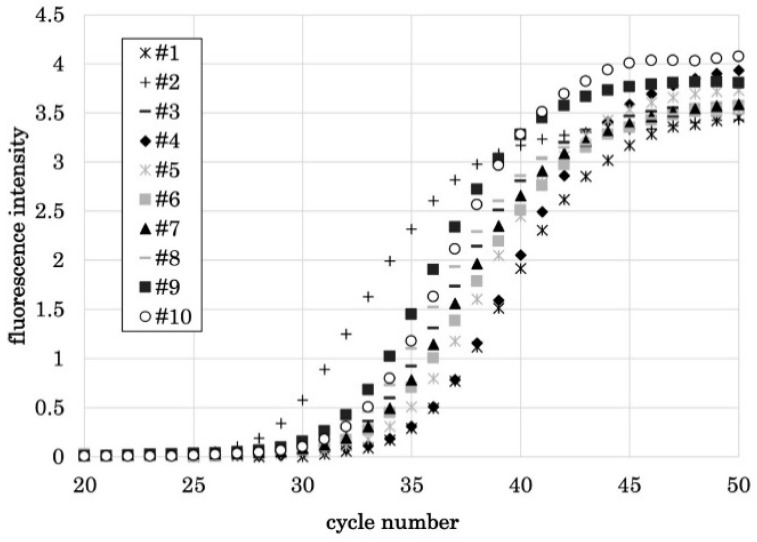
qPCR results of 10 subjects. Eight of the 10 subjects were able to perform qPCR for the first time without any problems. Two people were able to perform qPCR without any problem after a simple training. A stable result was obtained with an average Ct value of 27.56.

**Figure 4:**
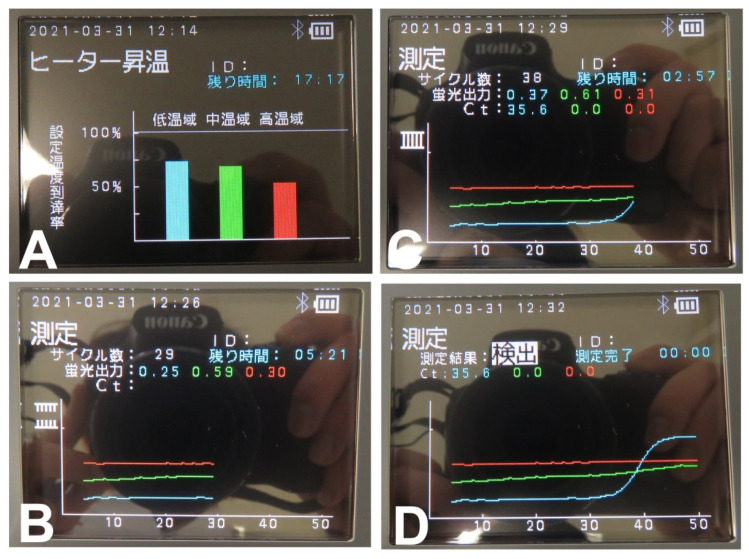
Screen display during qPCR with PCR1100: (**A**) The heat block heating up. (**B**) Amplification reaction. Three graphs are displayed as it progresses. Blue, RNase P; Green, CDC N1; Red, CDC N2. (**C**) The blue graph shows an increase (i.e., presence of RNA). The Ct value when the graph rises is displayed (here, it is 35.6). (**D**) The reaction continues up to 50 cycles. At the end of 50 cycles, the final measurement result and each Ct value are displayed.

Eight of the 10 participants were able to perform qPCR using the PCR1100 upon the first attempt without any problems. Although two of the participants produced an error upon their first attempt, they were able to carry out the steps error-free after receiving a brief re-explanation and additional pipette operation explanation (Figure 3). The screen display of the actual machine at that time is shown in Figure 4. The average Ct value of the 10 participants was 27.56, and stable results were obtained. Therefore, it was confirmed that with simple explanation and training, people who do not have knowledge or experience in molecular biology can use this device correctly.

### 3.3. qPCR Using a Trace Amount of Saliva as a Sample

RNase P synthetic RNA was mixed into saliva without any pretreatment, and PCR1100 performed qPCR using this mixture as a sample. At first, when saliva was used as a stock solution sample, the high viscosity hindered the movement of the sample in the flow path chip, and qPCR did not operate normally. However, when saliva was diluted two-fold, qPCR operated normally, and contaminated RNase P synthetic RNA was detected. In addition, to prove that this result was not caused by the saliva of a specific subject, the same procedure was performed using representative saliva mixed with the saliva of five subjects, and qPCR performed normally. As a result, it was confirmed that this device can perform qPCR using saliva without any pretreatment as a sample (Table 3).

Next, since saliva originally contains RNase P, it was examined whether it could be detected without adding RNase P synthetic RNA to the saliva sample in advance. As a result, it was possible to detect endogenous RNase P in saliva using two- or four-fold diluted saliva as a sample. Thus, it was confirmed that this device can detect RNA contained in saliva without pretreatment. However, since the Ct value exceeded 40 in the four-fold dilution, it was considered appropriate to dilute the saliva approximately two-fold (Table 4).

From the viewpoint of being simpler and reducing the risk of infection, it was examined whether a sample collected by the mouthwash method instead of the saliva spitting method could be used for the purpose of omitting the step of diluting saliva. As a result, it was confirmed that when a mouthwash with 1 or 2 mL of 10-fold diluted PBS was used as a sample for 10 or 15 s, endogenous RNase P in saliva could be detected using the mouthwash as a sample under any condition (Table 5). Furthermore, a mouthwash with physiological saline, which is easier to apply clinically, was used as a sample. As a result, it was confirmed that endogenous RNase P in saliva could be detected in mouthwash for 15 s with 2 mL of physiological saline (Table 5). From this, it was found that when clinically applying this device as a POCT, 2 mL of physiological saline for 15 s of mouthwash was an easy and stable method to use for sample collection. In addition, it was possible to detect endogenous RNase P in the frozen samples without any problems (Table 5).

Furthermore, to clarify factors that affect sample collection, we examined the samples at normal times, after meals, and after brushing. As a result, compared to normal times, diet was not affected and endogenous RNase P originally contained in saliva could be detected. As was detected after brushing, the Ct value tended to increase. It was confirmed that collecting a sample using this device should be avoided immediately after brushing. This result was the same for both diluted saliva and mouthwash (Table 6).

### 3.4. Detection of SARS-CoV-2 Synthetic RNA

We investigated whether this device could detect SARS-CoV-2 N1 and N2 synthetic RNA in saliva. As a control, when synthetic RNAs of RNase P, CDC N1, and CDC N2 were mixed with pure water and qPCR performed with the PCR1100, detection was successful. Thus, it was confirmed that there was no problem using the SARS-CoV-2 detection protocol set with this device. Therefore, SARS-CoV-2 N1 and N2 synthetic RNA were mixed with the mouthwash of four subjects, and qPCR was performed with the PCR1100 using this as a sample. As a result, it was confirmed that SARS-CoV-2 synthetic RNA can be detected using the mouthwash of any subject. From this, it was determined that mouthwash can be used as a sample for detecting SARS-CoV-2 with this device (Table 7). However, in the sample in which synthetic RNA was directly mixed with mouthwash, the Ct value at the time of detection tended to increase with the passage of time compared with the sample in which synthetic RNA was added to the premix in advance. This was attributed to the progress of RNA degradation by the saliva component, although detection was achieved with each of them (Table 7).

### 3.5. Detection of SARS-CoV-2 Viral RNA from COVID-19 Patients

**Figure 5:**
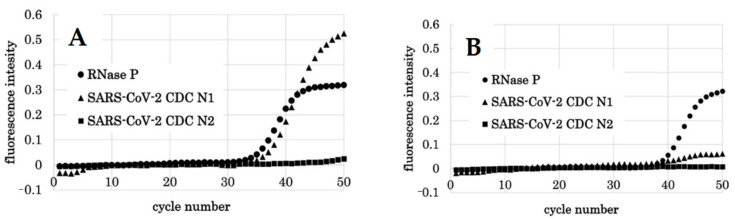
qPCR results by PCR1100 of subject 12. (**A**) Results of the first qPCR (8th day after the onset of symptoms). RNase P was detected with a Ct value of 34.4, indicating that there was no problem with the PCR reaction. CDC N1 increased at a Ct value of 35.7, and SARS-CoV-2 viral RNA was detected. At this time, CDC N2 was not detected. (**B**) Results of the second qPCR (15 days after the onset of symptoms). RNase P was detected with a Ct value of 38.4, indicating that there was no problem with the PCR reaction. CDC N1 was not detected.

**Figure 6:**
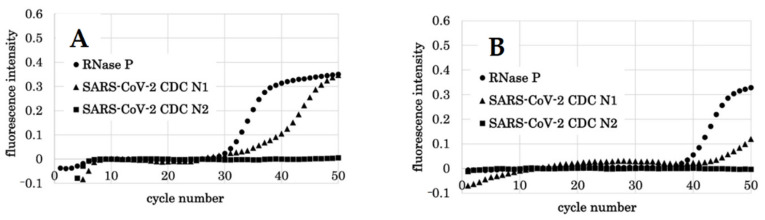
qPCR results by PCR1100 of subject 8. (**A**) Results of the first qPCR (3rd day after the onset of symptoms). RNase P was detected with a Ct value of 29.8, indicating that there was no problem with the PCR reaction. CDC N1 increased at a Ct value of 38.4, and SARS-CoV-2 viral RNA was detected. At this time, CDC N2 was not detected. (**B**) Results of the second qPCR (10 days after the onset of symptoms). RNase P was detected with a Ct value of 38.4, which indicated that there was no problem with the PCR reaction. CDC N1 was not detected. However, the graph showed an upward trend near 50 cycles.

We investigated whether SARS-CoV-2 viral RNA could be detected by this device using the mouthwash of COVID-19 patients who had been diagnosed as SARS-CoV-2 positive by the conventional qPCR test. Of the 20 participants who gave their consent, sampling was performed in 17 people and three were excluded due to the ambiguous date of the onset of symptoms. Initially, CDC N2 was not detected in all cases. This was considered to be a problem of sensitivity, which made it necessary to improve the concentration of primer and annealing temperature. In the future, adjustments should be made to improve the sensitivity of the test so that N1 and N2 can be detected to the same extent. As a result, CDC N1 could be detected without any problem in the subjects who had a short period from the onset of symptoms. This period was approximately 10 days (Table 8). As a typical case, in subject 12, SARS-CoV-2 virus RNA was detected with a Ct value of 35.7 on the 8th day but was not detected on the 15th day (Figure 5). In subject 8, the Ct value was detected at 38.4 on the 3rd day but was not detected on the 10th day (Figure 6). However, looking at the graph on the 10th day, an upward trend was observed in the graph near 50 cycles. It was presumed that a small amount of virus was present in the mouthwash around 10 days, which was considered to be proof that the detection limit was around 10 days. For all subjects, there was a tendency that the virus could not be detected in the mouthwash samples after ~10 days. However, there were cases, such as subject 2, in which detection was possible after 10 days even if the period from onset of symptoms was long (Table 8).

## 4. Discussion

Pandemic control of COVID-19 includes preventative measures, such as social distancing, wearing masks, and maintaining good hand hygiene, but as a complement, isolation of positive cases and extensive population screening are important preventative measures [1]. However, SARS-CoV-2 virus RNA detection by conventional RT-qPCR techniques, such as using nasopharyngeal swab samples, are costly, have a long turnaround time, require specialized personnel, and might require a device that is unavailable. Therefore, extensive screening tests have not been conducted [4]. However, in some countries, such as China, the qPCR test is being used for screening. However, it should be taken into consideration that the actual conditions of testing differ greatly between countries, and the infection status and the degree of containment aimed at are also different. Since there is no debate about the superiority of the qPCR test itself, it is significant that various qPCR devices and sample collection methods exist as options. If it can be selected according to the situation of each country, it is considered that more effective infection control measures against the SARS-CoV-2 pandemic can be achieved. An antigen test is an alternative test method to the current qPCR test. This test is user-friendly, low-cost, quick, and utilizes a small test device, so it can be widely available [7]. However, the main disadvantage is that the sensitivity is lower than that of the qPCR test, which makes it less suitable as a screening test [8,9]. In the future, it is expected that mutations in the virus will occur. At this time, there is a possibility that the mutant strain cannot be dealt with by the antigen test applying the antigen-antibody reaction. However, if it is qPCR, it can be applied immediately by setting a new primer in the mutant part. From this, it is considered that the qPCR method is highly useful in the future. Recently, the development of a LAMP test using saliva as a sample [10] and an attempt to place a satellite laboratory in the inpatient ward of a COVID-19 patient to perform a rapid test [11] have been reported. Various other test methods have been developed and studied [12,13,14,15,16], but none have achieved the same sensitivity and specificity as the conventional qPCR test. Recently, POCT is a test concept that has been attracting attention, as several small devices that can perform qPCR in a short time have been developed, which can overcome many of the aforementioned problems. Attempts to apply these as SARS-CoV-2 detection devices are being reported [17]. The introduction of POCT equipment is cost-effective, provides detection capabilities that are comparable to conventional qPCR equipment, and eliminates the need for large laboratories and equipment. It is very useful and suitable as a screening test at the time of a pandemic such as COVID-19. Therefore, since January 2020, various researchers and companies have focused on the development of POCT testing devices for rapid diagnosis of SARS-CoV-2. As mentioned earlier, several types of POCT devices have been developed; they have achieved antibody detection [18,19,20,21,22,23], antigen detection [24,25,26], and nucleic acid detection [27,28,29]. The PCR1100 used in this study is a POCT device that focuses on nucleic acid detection, and as revealed in this study, it is user-friendly and can be performed easily, even by medical staff who have no knowledge or experience in molecular cell biology. Thus, it was proven that qPCR testing can be performed in a short time by using this device, even in hospitals and small clinics regardless of the practice (i.e., dentistry, medicine), without skilled clinical laboratory technicians and specialized staff.

On the other hand, the currently used sample collection method, a nasopharyngeal swab, poses the risk of infection due to the droplets at the time of collection, and the quality of the sample varies depending on the collection technique [2,3,30,31,32,33]. In order to overcome these problems, various body fluids, such as saliva, tears, and so on, have been verified to be effective samples for the purpose of facilitating safer sampling protocol [34,35,36,37,38,39,40,41]. Among these alternative sample types, saliva has been reported to be as sensitive and specific as nasopharyngeal swabs compared to other body fluids [42,43]. In this study, with the aim to develop a safer yet simpler, easy-to-use diagnostic method using the PCR1100 as a POCT device, saliva was used as a sample. As a result, when the undiluted saliva was used as a sample, its viscosity became a problem, and its movement in the flow path chip was hindered; therefore, the device did not operate normally. However, by diluting the saliva more than two-fold, the device operated normally, and RNase P synthetic RNA mixed in saliva could be detected. However, when the saliva was diluted four-fold or more, the Ct value at the time of detection tended to increase, so it was confirmed that dilution of approximately two-fold is appropriate. We also examined whether RNA of RNase P originally contained in saliva could be detected and found that endogenous RNase P could be detected without any pretreatment. Furthermore, to eliminate the step of diluting saliva and thus enable a simpler and safer sample collection, a 15-s mouthwash with 2 mL of physiological saline was tried as a sample. As a result, it was confirmed that endogenous RNase P could be detected. Moreover, this mouthwash could be used directly as a sample without any pretreatment. However, it was also confirmed that the timing of mouthwash collection impacted the sensitivity of detection; testing should be avoided immediately after brushing, although the influence of eating food is small.

Finally, we examined whether SARS-CoV-2 viral RNA could be detected using the mouthwash of a COVID-19 patient as a sample. SARS-CoV-2 synthetic RNA mixed into the mouthwash could be detected under the conditions established so far without any problem. Therefore, when this diagnostic method was performed on patients undergoing hospitalization under the diagnosis of COVID-19 using mouthwash as a sample, SARS-CoV-2 virus RNA could be detected without any problem. In addition, the detection of SARS-CoV-2 viral RNA depends on the period from onset of symptoms. It was confirmed that SARS-CoV-2 viral RNA can be detected using this device. As was reported in previous reports [42,43,44], the detection results when using mouthwash suggested that there is a detection limit depending on the period from the onset of symptoms, which is around 10 days. It is necessary to keep this in mind when using PCR1100 as a POCT device and in future screenings, but it was found that the device’s performance was sufficient for achieving the intended purpose. Table 9 summarizes the advantages of PCR 1100 over current qPCR devices. In this study, results from PCR 1100 could not be compared with those from conventional qPCR devices under current Japanese infection control regulations. However, it was found that the method using PCR 1100 could detect SARS-CoV-2 in a mouthwash sample containing a small amount of saliva. In order to verify the detection accuracy of PCR 1100, the number of samples will be increased and compared with the results obtained using conventional qPCR, which will be reported in the near future. In addition, although we did not limit the Ct value at the time of N1 detection this time, in the future, it will be necessary to adjust the Ct value for diagnosing SARS-CoV-2 positivity. In addition, N2 could not be detected at all in this study. This was considered to be a problem of sensitivity, which can likely be resolved by as adjustments to certain settings such as the primer concentration and annealing temperature. In the future, we think it will be necessary to find a setting that can detect N1 and N2 to the same extent. Improvement of N2 detection conditions is now nearly complete and is in the process of final confirmation for actual COVID-19 patients. We plan to release the data in the near future. Moreover, by performing this test frequently in the future, it may be possible to utilize it not only for the detection of SARS-CoV-2 virus, but also for the determining the course of treatment and discharge time. Either way, as a screening tests, this method, which can quickly and easily diagnose the presence or absence of SARS-CoV-2 viral RNA contained in saliva, can promote more extensive testing in small clinics and contribute to infection control [44,45,46]. This diagnostic method is an effective screening test that supports the early diagnosis and management of COVID-19 infection.

The possibility of spreading COVID-19 infection via saliva aerosol has been pointed out, and medical practice where saliva aerosol is generated, especially in dentistry, has severely restricted the medical care provided, thus causing serious public health problems [47,48]. If this diagnostic method can be applied, performing a screening test before providing outpatient treatment will become easier in small clinics and dental clinics. This result makes it possible to implement an infection control measure that enables health care professionals to perform treatments that generate aerosols with less risk of spreading COVID-19. Successful infection control in the future depends on effective measures such easy-to-use screening methods with a rapid turnaround time that can be operated in small clinics without specialized laboratory equipment and technicians. We are confident that this diagnostic method will greatly contribute to the establishment of new trends in screening for better infection control in the future.

## 5. Conclusions

This diagnostic method is a simple and rapid qPCR test that produces accurate results from the mouthwash of COVID-19 patients. In addition to its efficacy and rapid results, this diagnostic method is easy to use and cost effective, which makes it practical for small clinics without specialized technicians to implement this screening test to minimize the risk of the virus’ spread. In future studies, we plan to increase the number of cases tested at multiple centers to confirm the accuracy of this diagnostic method.

## Figures and Tables

**Table 1:** PCR primers/probes and control RNA sequence.

Target Name		Sequence	Final Concentration	Amplicon
hRNase P	forward reverse probe	5′-AGA TTT GGA CCT GCG AGC G-3′ 5′-GAG CGG CTG TCT CCA CAA GT-3′ 5′-FAM-TTC TGA CCT GAA GGC TCT GCG CG-BHQ1-3′	0.40 μM	65 bp
			0.40 μM	
			0.20 μM	
2019-nCoV_Nl (CDC N1)	forward reverse probe	5′-GAC CCC AAA ATC AGC GAA AT-3′ 5′-TCT GGT TAC TGC CAG TTG AAT CTG-3′ 5′-HEX-ACC CCG CAT TAC GTT TGG TGG ACC-BHQ1-3′	0.20 μM	72 bp
			0.20 μM	
			0.20 μM	
2019-nCoV_N2 (CDC N2)	forward reverse probe	5′-TTA CAA ACA TTG GCC GCA AA-3′ 5′-GCG CGA CAT TCC GAA GAA-3′ 5′-Cy5-ACA ATT TGC CCC CAG CGC TTC AG-BHQ3-3′	0.40 μM	67 bp
			0.40 μM	
			0.20 μM	
**Control RNA**	**Sequnce**			
hRNase P (ll0 bp)	GGACUUCAGCAUGGCGGUGUUUGCAGAUUUGGACCUGCGAGCGGGUUCUGACCUGAAGGCUCUGCGCGGACUUGUGGAGACAGCCGCUCAUUGUGAGUUGCCCCGGCUUC			
Nl (120 bp)	ACAAACUAAAAUGUCUGAUAAUGGACCCCAAAAUCAGCGAAAUGCACCCCGCAUUACGUUUGGUGGACCCUCAGAUUCAACUGGCAGUAACCAGAAUGGAGAACGCAGUGGGGCGCGAUC			
N2 (200 bp)	UGGUCCAGAACAAACCCAAGGGAAAUUUUGGGGACCAGGAACUAAUCAGACAAGGAACUGAUUACAAACAUUGGCCGCAAAUUGCACAAUUUGCCCCCAGCGCUUCAGCGUUCUUCGGAAUGUCGCGCAUUGGCAUGGAAGUCACACCUUCGGGAACGUGGUUGACCUACACAGGUGCCAUCAAAUUGGAUGACAAAGAUC			

The fluorescent dyes attached with the 5′ end of each probe were as follows: 6-carboxyfluorescein (FAM) for N_Sarbeco_P; cyan5 (Cy5) for NIID_2019-nCOV_N_P2; FAM or 6-carboxy-2′,4,4′,5′,7,7′-hexachlorofluorescein (HEX) for 2019-nCoV_N1-P; HEX or Cy5 for 2019-nCoV_N2-P; FAM for HCoV-229E_NP; FAM for RNase P. Following the CDC protocol, detection of SARS-CoV-2 was performed with multichannel 2019-nCoV_N1-P with FAM and N2-P with HEX were utilized, respectively. However, CDC protocol for RNase P detection was performed with multichannel, N1-P with HEX and N2-P with Cy5. All probes were attached with Black Hole Quencher (BHQ, Biosearch Technologies, Inc., Petaluma, CA, USA) at the 3′ end.

**Table 2:** The subjects’ occupation and age.

Number	Occupation	Age
1	Dental hygienist	43
2	Dental hygienist	26
3	Dental hygienist	29
4	Nurse	47
5	Dental hygienist	36
6	Nurse	53
7	Dental hygienist	37
8	Nurse	45
9	Nurse	49
10	Nurse	44

**Table 3:** qPCR results when using saliva mixed with RNase P synthetic RNA as a sample.

Sample	Detection Status	Ct Value
Saliva (undiluted solution)	Not detected	-
Saliva (diluted 2-fold with pure water)	Detected	23.34
Representative saliva (for 5 people) (diluted 2-fold with pure water)	Detected	28.50

**Table 4:** qPCR results using saliva (without RNase P synthetic RNA contamination).

Sample	Detection Status	Ct Value
Saliva (diluted 2-fold with PBS)	Detected	36.08
Saliva (diluted 4-fold with PBS)	Detected	42.34

**Table 5:** qPCR results when mouthwash is used as a sample.

Sample (Collect 3 µL from Mouthwash and Perform qPCR)					Detection Status	Ct Value
Wash	mouth	with	1 mL	of 10-fold diluted PBS for 10 s	Detected	37.30
Wash	mouth	with	2 mL	of 10-fold diluted PBS for 10 s	Detected	38.30
Wash	mouth	with	1 mL	of 10-fold diluted PBS for 15 s	Detected	34.43
Wash	mouth	with	2 mL	of 10-fold diluted PBS for 15 s	Detected	33.63
Wash	mouth	with	2 mL	of saline for 15 s (subject 1)	Detected	37.74
Wash	mouth	with	2 mL	of saline for 15 s (subject 2)	Detected	39.03
Wash	mouth	with	2 mL	of saline for 15 s (subject 3)	Detected	35.76
Wash	mouth	with	2 mL	of saline for 15 s (subject 1)	Detected	35.45
				(After freezing for 2 weeks)		
Wash	mouth	with	2 mL	of saline for 15 s (subject 2)	Detected	37.98
				(After freezing for 2 weeks)		
Wash	mouth	with	2 mL	of saline for 15 s (subject 3)	Detected	35.14
				(After freezing for 2 weeks)		

**Table 6:** Effect of diet and brushing (Ct value).

Conditions for Sample Collection	Diluted Saliva		Mouthwash
	2-Fold with PBS	4-Fold with PBS	
Normal time (3 h after eating and brushing)	36.08	42.34	37.12
After meals (30 min)	39.44	38.63	37.54
After brushing (15 min)	42.15	40.60	41.15

**Table 7:** Detection of SARS-CoV-2 synthetic RNA (CDC IM1, CDC IM2) mixed in saliva samples.

Sample	Conditions	Ct Value		
		RNase P	CDC Nl	CDC N2
Positive control	Synthetic RNA of 10^7^ copies each in pure water	21.09	18.95	24.74
Subject 1	Premix containing synthetic RNA+ Mouthwash 3 µL	38.40	18.96	37.54
Subject 1	Premix + Mouthwash 3 µL containing synthetic RNA	33.97	31.44	>50
Subject 2	Premix containing synthetic RNA+ Mouthwash 3 µL	36.99	19.61	26.37
Subject 2	Premix + Mouthwash 3 µL containing synthetic RNA	33.19	26.27	43.83
Subject 3	Premix containing synthetic RNA+ Mouthwash 3 µL	35.62	19.92	26.87
Subject 3	Premix + Mouthwash 3 µL containing synthetic RNA	32.39	27.50	43.59
Subject 4	Premix containing synthetic RNA+ Mouthwash 3 µL	39.84	18.68	25.92
Subject 4	Premix + Mouthwash 3 µL containing synthetic RNA	36.36	26.60	42.41

**Table 8:** Detection of SARS-CoV-2 viral RNA (CDC Nl) in COVID-19 patients by PCR1100.

Subjects	Period from Onset of Symptom (Days)/Detect (Ct Value) or Not (-)			Symptom (Period, Days)	Past History
Age Sex	1st	2nd	3rd		
1. 38 M	13/44.6	20/(-)		Fever (6), malaise, dysgeusia	HT, DM, HL, CCF
2. 90 M	7/29.3	14/34.5	21/38.8	Fever (8), breathing difficulty	HT, DM, CKD
3. 56 F	18/(-)	25/(-)		Fever, respiratory management (7)	n.p.
4. 34 M	ll/(-)			Fever (10), dysgeusia	n.p.
5. 34 F	7/38.1	14/(-)		Fever (5), breathing difficulty	DM, HT, obesity
6. 47 F	ll/(-)	18/(-)		Fever (11), dysgeusia	CKD, thyroid and parathyroid cancer
7. 67 M	Exclusion				
8. 47 M	3/38.4	10/(-)		Fever (5), cough	CKD, HD, DM
9. 76 M	9/45.2			Fever (7), breathing difficulty	n.p.
10. 44 M	7/36.9	14/(-)		Fever (10), breathing difficulty	DM, HT, HL
11. 30 M	10/(-)			Fever (10), breathing difficulty	n.p.
12. 22 M	8/35.7	15/(-)		Fever (l0), diarrhea	n.p.
13. 57 M	10/(-)			cough, breathing difficulty	DM
14. 61 M	8/34.2			Fever (13), dysgeusia	integration disorder syndrome
15. 61 M	25/(-)			Fever (15), breathing difficulty	DM, HT
16. 34 M	11/34.1			Fever (11), breathing difficulty	DM
17. 61 M	Exclusion				
18. 54 M	Exclusion				
19. 81 M	1/40.2	8/37.7		Fever (1), pneumonia	HT *1
20. 60 F	5/40.9	12/(-)		Fever (4), breathing difficulty	CKD, HD

HT: hypertension, DM: diabetes mellitus, HL: hyperlipemia, CCF: chronic cardiac failure, CKD: chronic kidney disease, and HD: hemodialysis. *1: first vaccination 5 days before onset of symptoms.

**Table 9:** Advantages of the PCR1100 device compared with conventional qPCR devices.

	PCR1100	Conventional qPCR Device
Turnaround time	approximately 18 min	a few hours
Sample	mouthwash	nasopharyngeal swab, saliva, etc.
Person who carries out	skilled specialist, clinical laboratory technician	anyone
Ease of handling	mobile (POCT)	stationary type (cannot move)

## Data Availability

The data that support the findings of this study are available from the corresponding author upon reasonable request.

## Supplementary Materials

The following are available online at https://www.mdpi.com/article/10.3390/diagnostics11112024/s1.
