# Supplementary material for: Feasibility of Rapid Diagnostic Technology for SARS-CoV-2 Virus Using a Trace Amount of Saliva
Source: Diagnostics (Basel). 2021 Nov 1;11(11):2024. doi: 10.3390/diagnostics11112024 (PMC8625231; doi:10.3390/diagnostics11112024)
Supplement: Supplementary file 1 [file diagnostics-11-02024-s001.zip › diagnostics-1432177-supplementary.pdf]

diagnostics

Supplementary Materials

Examination of detecting synthetic RNA by multichannel and of detecting coronavirus in saliva: In a preliminary study, we investigated whether SARS-CoV-2 synthetic RNA could be detected utilizing this device. The result showed that this device combined with the CDC protocol could be detected SARS-CoV-2 synthetic RNA at high correlation of concentrations with Ct value. In addition, it was demonstrated that the human coronavirus 229E in saliva could be detected by direct real-time RT-PCR. (DOI: 10.1101/2021.05.01.21256441).
